# Acute Epstein-Barr Virus Infection Complicated by Rhabdomyolysis: A Case Report and Literature Review

**DOI:** 10.7759/cureus.29784

**Published:** 2022-09-30

**Authors:** Mohannad Faisal, Amr Ashour, Israa Alshahwani, Mas Chaponda

**Affiliations:** 1 Internal Medicine Department, Hamad Medical Corporation, Doha, QAT; 2 Infectious Diseases Department, Hamad Medical Corporation, Doha, QAT

**Keywords:** general internal medicine, clinical infectious medicine, clinical virology, rhabdomyolysis, primary ebv infection

## Abstract

Infectious mononucleosis is typically a self-limiting illness though it can cause serious complications. On the other hand, rhabdomyolysis can activate after intense exertion, drugs, or infections. We present a report of a patient who presented with infectious mononucleosis and developed rhabdomyolysis that was complicated by acute kidney injury requiring hemodialysis, which makes it the eighth most reported case worldwide according to our review. Our aim is to bring the attention of clinicians to the fact that serious complications like rhabdomyolysis can happen following such a common viral infection.

## Introduction

Infectious mononucleosis (IM) is an acute infectious disease consisting of fever, cervical lymphadenopathy, and pharyngitis accompanied by the presence of atypical blood lymphocytes [1]. Most cases of IM are usually caused by Epstein-Barr Virus (EBV). Other infectious agents, such as cytomegalovirus, can cause similar syndromes. EBV infection usually happens in children and young adults. The infection is spread primarily by close contact or oral secretions [2,3]. The most frequent signs and symptoms are a sore throat (95%), cervical lymphadenopathy (80%), fatigue (70%), upper respiratory symptoms (65%), headache (50%), decreased appetite (50%), fever (47%), and myalgias (45%) [2]. The diagnosis of EBV infection usually depends on clinical symptoms supported by serological tests. EBV infection is typically mild and self-limiting, but serious complications, such as airway obstruction, meningoencephalitis, hemolytic anemia, and thrombocytopenia can occur in at least 1% of cases. Other complications, such as splenic rupture, occur in less than 1% of cases [4].

Rhabdomyolysis is a medical condition involving the rapid dissolution of damaged or injured skeletal muscles. This disruption of skeletal muscle integrity leads to the direct release of intracellular muscle components, including myoglobin, creatine kinase (CK), aldolase, and lactate dehydrogenase, as well as electrolytes, into the bloodstream [5]. Rhabdomyolysis can present as a spectrum from subclinical elevation in the CK level to a life-threatening condition associated with electrolyte imbalance, acute renal failure (ARF), and disseminated intravascular coagulation [6]. Rhabdomyolysis is mostly caused by direct injury but can also result from exposure to drugs, toxins, infections, muscle ischemia, electrolyte and metabolic disorders, genetic disorders, and excessive exertion [7].

In this study, we present data from a patient with acute EBV infection complicated by rhabdomyolysis with a review of the reported studies about the possible association.

## Case presentation

A 16-year-old male patient presented to the emergency department with increasing fatigue and generalized body pain. He was seen one week earlier in the primary health care center (PHCC) with fever, malaise, and sore throat with pharyngeal erythema. Symptomatic treatment for tonsilitis was given. He was seen three days later in the PHCC. The patient reported a resolution of his fever but was still complaining of fatigue. On the day of admission, he had severe myalgia and fatigue, making him unable to leave his bed. He reported a loss of appetite, nausea, and vomiting. He reported passing dark-colored urine with no dysuria, urgency, or frequency. He did not smoke or drink any alcohol. He denied illicit drug use, intense physical exertion, prolonged sun exposure, or trauma. He had a past medical history of Glucose 6-phosphate dehydrogenase (G6PD) deficiency that was discovered at neonatal screening, but he had no prior hemolysis episodes.

On examination, his temperature was afebrile, pulse rate 84 beats/minute, blood pressure 105/70 mmHg, and respiratory rate 18 per minute. He had no peripheral edema or lymphadenopathy. His abdomen was not tender and there was no organomegaly. The rest of the systemic examination was unremarkable.

The laboratory investigations on presentation showed raised serum creatinine (Cr) 939 µmol/L; urine dipstick indicated +++ blood with urine RBC 18 per HPF; hemoglobin 10 g/dL; platelets 446000/µL; myoglobin 23779 ng/mL; and creatine kinase > 4000 U/l (Table 1). The serum electrolytes panel showed raised serum potassium and phosphorus with low serum calcium.

**Table 1:** Biochemistry LDH: lactate dehydrogenase; AST: aspartate aminotransferase; ALT: alanine transaminase

Laboratory test	Results	Normal ranges
Hemoglobin	10.1	(13-17) g/dl
White blood cells	24000	4000-1000 cell/µL
Platelet	446000	150000-450000/µL
Blood urea	59	(2.5-7.8) mmol/L
Serum creatinine	939	(54-95) µmol/L
Serum sodium	118	(133-146) mmol/L
Serum potassium	5.9	(3.5-5.3) mmol/L
Serum phosphate	3.55	(0.8-1.5) mmol/L
Corrected serum calcium	1.55	(2.2-2.6) mmol/L
Serum bicarbonate	23.7	(22-29) mmol/L
Serum magnesium	1.15	(0.7-1) mmol/L
Creatine kinase	>4000	(34-147) U/L
Myoglobin	23.779	(28-72) ng/mL
LDH	>2400	(130-250) U/L
ALT	643	(9-24)U/L
AST	1413	(13-26) U/L
Bilirubin total	28.9	(3.4-20.5) µmol/L
Alkaline phosphatase	75	(89-365) U/L

Urine studies showed urine sodium <20 mmol/L (Table 2).

**Table 2:** Urine studies

Laboratory test	Results
Urine WBC	215/HPF
Urine RBC	18/HPF
Urine dipstick for blood	+++
Urine sodium	<20 mmol/L
Urine protein/creatinine ratio	142 (normal<22 mg/mmol)
toxicology	negative

**Figure 1:**
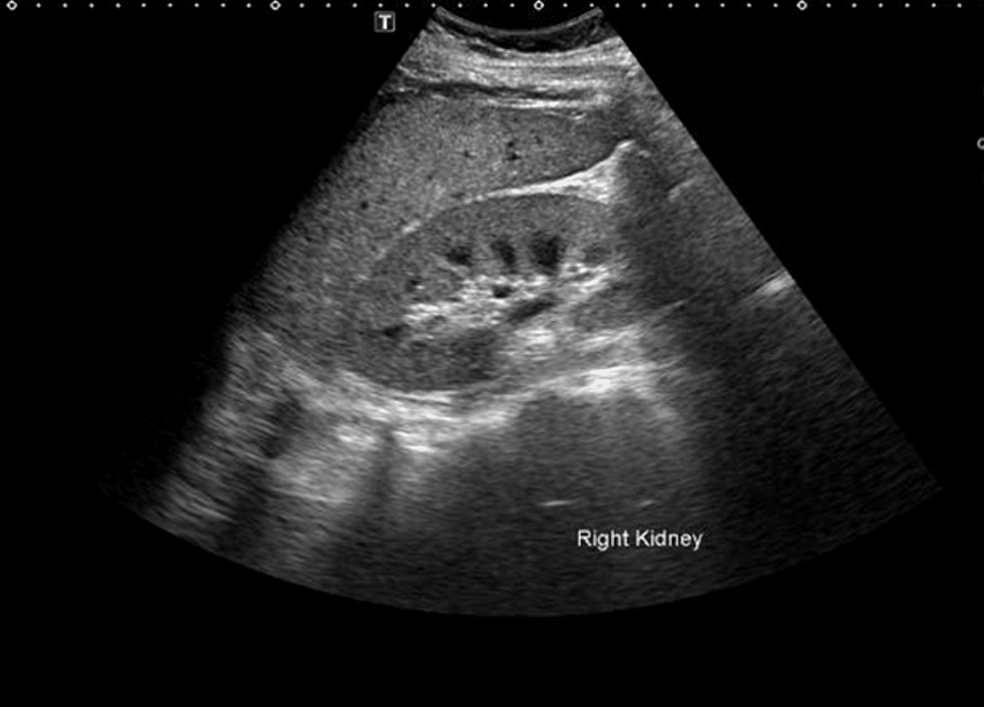
Ultrasound of the right kidney Ultrasound shows an increase in renal cortical echogenicity. Kidney size and cortical thickness are preserved.

Ultrasound of the kidneys showed normal-sized kidneys and no signs of obstructive uropathy (Figure 1).

The results of liver function tests are as follows: alanine aminotransferase (ALT) of 634 U/L, aspartate aminotransferase (AST) of 1413 U/L, alkaline phosphatase of 75 U/L, and total bilirubin of 28.9 µmol/L. Blood cultures were negative, and HIV and hepatitis serology were negative. The autoimmune screening panel as shown in Table 3.

**Table 3:** Serology and autoimmune workup

Laboratory test	Results
EBV IgG	positive
EBV IgM	positive
Antinuclear antibody	negative
Anti-double stranded antibody	negative
ANCA	negative
HIV	negative
CMV	negative
Hepatitis virus serology	negative

C3 and C4 were normal, with a negative test for antineutrophil cytoplasmic antibodies (ANCA). His Epstein-Barr virus (EBV) capsid immunoglobulin M (IgM) and (IgG) were positive while the EBV polymerase chain reaction was negative. The peripheral smear showed hypochromic microcytic anemia with no schistocytes or bite or blister cells. There were no atypical lymphocytes. His G6PD enzyme level was low (61%).

The initial diagnosis was acute kidney injury (AKI) secondary to rhabdomyolysis, with acute EBV infection as the cause of infectious mononucleosis (IM).

The patient was managed initially with intravenous fluid but developed oliguria and fluid overload. As a result, hemodialysis was initiated. He became better and his liver function improved.

During his hospital stay, his haemoglobin started to decline, reaching (5.8) gm/dL. No bleeding source was found. A blood test for hemolytic anemia revealed a low haptoglobin level, an increase in total and direct bilirubin levels, and an improvement in ALT and AST. The peripheral smear was repeated and showed blister cells. The direct antiglobulin test was negative. The patient was diagnosed with acute hemolysis due to G6PD deficiency, which was managed by supportive care and blood transfusion.

After two weeks of presentation, the patient's urine output and renal function tests began to improve, and he was discharged home three weeks later without the need for hemodialysis. He was followed two weeks after discharge from the clinic. He was clinically fine, and his creatinine was 93 µmol/L.

## Discussion

Various types of infections have been suggested to be possible causes of rhabdomyolysis. Influenza viruses are the most common viruses to be associated with rhabdomyolysis [8,9] while other viruses have also been implicated. Bacterial Infection can also cause rhabdomyolysis either by direct infection or due to sepsis without direct muscle injury [5].

Here, we report a young man with infectious mononucleosis complicated by rhabdomyolysis and acute kidney injury, which required hemodialysis. Acute EBV infection was diagnosed on clinical presentation supported by positive serum IgM and IgG. However, EBV PCR in blood was negative. We attributed that to the short life of viremia in acute EBV infection in immunocompetent patients like our patient [10].

Although various mechanisms of kidney involvement have been reported as a consequence of EBV infection [11], we think that the kidney involvement in our case was due to the tubular injury as a result of rhabdomyolysis in the context of the typical clinical and laboratory picture of rhabdomyolysis.

On review of the literature, we found seven reported cases of such an association [12-18]. Of these, three patients developed rhabdomyolysis without kidney involvement while in four cases, acute kidney injury was present along with rhabdomyolysis [12-18].

Potential confounders were present in some of the reported cases. McCabe et al. reported infectious mononucleosis complicated by rhabdomyolysis, but their patient had intense physical exertion in the preceding days of admission, unlike our case [12]. In the case reported by Aloizos et al., the patient was in the intensive care unit for airway obstruction and exposed to an anesthetic agent, which may be the trigger of rhabdomyolysis [16]. In the case reported by Poels et al., echovirus was isolated from muscle biopsy along with serological evidence of acute EBV infection [18], while in the other cases [13-15,17] we didn’t find other potential causes of rhabdomyolysis. One can argue here that acute hemolysis due to G6PD may have precipitated AKI in our patient, but our patient had no evidence of hemolysis on presentation when kidney injury was established, and acute hemolysis developed later in the course of the illness.

## Conclusions

Infectious mononucleosis due to acute EBV virus infection could be associated with acute rhabdomyolysis although further studies are needed to prove the causality between the two diseases. Clinicians should keep in mind that serious complications can happen following infectious mononucleosis with proper referral to tertiary hospitals if such complications are suspected.
